# Concise ethnobotanical review and botanical pharmacognosy of turmeric rhizome (*Curcuma longa* L.)

**DOI:** 10.1080/13880209.2026.2740863

**Published:** 2026-10-14

**Authors:** Roy T. Upton

**Affiliations:** American Herbal Pharmacopoeia, Scotts Valley, CA, USA

**Keywords:** Turmeric, curcuma, ethnobotany, pharmacognosy

## Abstract

**Context:**

Turmeric (*Curcuma longa* L. Zingiberaceae) is a highly valued botanical medicine worldwide and has emerged as among the best-selling herbal products in North America.

**Objective:**

Presented an ethnobotanical record of turmeric use to present a context for this edition on turmeric.

**Methods:**

Information was primarily compiled from online sources including PubMed, Web of Science, Google Scholar, and Science Direct using ‘turmeric’, ‘curcuma’, and ‘curcuma longa’ as search terms; No time limit was specified. The primary goal was to mine ethnobotanical literature regarding turmeric use of various cultures. Online searches were augmented by hardcopy searches of ethnobotanical, and pharmacognosy literature from the first century.

**Results and conclusion:**

Turmeric is used in an array of preparations. It is listed among the top ten spices in the world and is used as a dye, medicine, object of cultural and religious significance, and coloring agent. Turmeric is used in African, Ayurvedic, Korean, Malaysian, Siddha, Chinese, Persian, Unani, and Western herbal traditions though India remains the largest producer and consumer of turmeric. Turmeric is universally used to support healthy gastrointestinal, circulatory, respiratory, and renal systems. The treatment of jaundice was one of turmeric’s primary historical indications. In combination with other botanicals, turmeric is used for treating almost any condition. Following is an ethnobotanical review of its traditional uses and select botanical pharmacognostic characteristics used for ensuring authenticity of raw material, including a discussion of some of the most common adulterants of turmeric products.

## Introduction

Turmeric is the common name of the rhizome (commonly referred to as turmeric root) and plant of *Curcuma longa* L., a member of the Zingiberaceae that include other important botanicals such as ginger, galangal, and cardamom. Turmeric powder is widely recognized as the primary ingredient of orange-colored curry. There is archeological evidence of turmeric use spanning 4,000 years. Turmeric is used in healing traditions worldwide and is one of the most important botanical medicines of formalized medical systems with India being the largest commercial producer and consumer of turmeric and among the most extensively developed ethnobotanical record of use.

## Nomenclature

**Table:** 

Standard Common Name:	Turmeric
Botanical Name:	Curcuma longa L. (synonym Curcuma domestica Valeton)
Family:	Zingiberaceae
Part Used:	Rhizome

### Other Names

Arabic: Kurkum

Assamese: Lidar, halodhi

Bengali: Halud

Burmese: Tanum

Cambodian: Ro miet

Chinese: Jiang huang (lateral roots); yu jin (tubers)

Danish: Gurkemeje

Dutch: Geelworte

Filipino (Tagalog): Dilau, luyang-dilau

French: Curcuma, safran des Indes

German: Kurkuma, Gelbwurzel

Gujurati: Haldar, haladhar

Hindi: Haldi, haldee

Indonesian: Kunik, dilao

Italian: Curcuma, zafferano delle Indie, turmerico

Japanese: Ukon

Kannada: Arishina, arisina, arisiya

Konkani: Halad

Laotian: Khi min

Maley: Kooneit

Malayalam: Manjal

Marathi: Halad, halde

Oriya: Haldi

Nepali: Besar

Norwegian: Gurkmeie

Oriya: Haladi

Portuguese: Acafrao-da-India

Punjabi: Haldar, haladhar, haldi

Russian: Kуркума (curcuma), жёлтый имбирь (yellow ginger)

Spanish: Curcuma

Sanskrit: Gauti, varnavat, haridra, marmadri, nisha, shati, haldi, harita

Swedish: Gurkmeja

Tamil: Manjal

Telegu: Pasupu, haridra

Thai: Kha min

Ukraine: Kуркума

Urdu: Haldi

Vietnamese: Nghệ, uất kim

### Botanical description

*Curcuma longa* L. is a perennial, rhizomatous, herbaceous sterile monocot of the ginger family (Zingiberaceae) believed to be native to India and Southeast Asia and occurring predominantly in the plains. The greatest diversity of the genus occurs in India, Myanmar, and Thailand. Due to its long-term cultivation turmeric is often referred to as *C. domestica*. The family is characterized by their distinctively rhizomatous horizontal underground stems, which are the primary plant part used.

*Curcuma* L. is a taxonomically difficult genus to identify due to the close resemblance of the vegetative parts and short flowering period of many *Curcuma* species (Skornickova et al. 2008). There are 20 species available in southern India; of these, 10 lack prominent rhizomes (Seema 2015; Anu et al. 2020).

Turmeric is widely cultivated throughout the world with the majority (80–90%) of the world’s production in India where approximately 70 cultivars specific to various regions are identified. Hybridization of *C. longa* with other species (e.g., *C. aromatica*) can occur (Nair 2019). There are six taxonomic varieties with *C. longa* var. *typica* and *C. longa* var. *atypica* predominating in India (see Table 1).

**Table 1.** Taxonomic varieties of turmeric (*Curcuma longa*).

*C. longa* var.* typica*	*C. longa* var *atypica*	*C. longa* var.* camphora*
*C. longa* var *spiralifolia*	*C. longa* *musacifolia*	*C. longa* var. *platifolia*

Source: Touwaide and Appetiti (2023).

Turmeric plants grow to 1 m tall. The leaves are petiolate encased in sheaths consisting of 6–10 leaves per sheath basally attached to the pseudostem, are alternately arranged, large and highly varied (∼30 cm long *x* ∼15 cm wide), lanceolate or oblong, rarely narrowly linear with an entire margin, prominent midvein and parallel veins typical of monocotyledon plants, and are moderately to deep green in color. The inflorescence emerges from the rhizome sometimes prior to or through the center of the leaves on a singular erect peduncle forming a spike of numerous bracteal imbricated scales between which the flesh- to pinkish-colored flowers emerge, spreading at the free end. The corolla is monopetalous consisting of a narrow tube, divided at the mouth into three oval segments with short filaments, four of which are erect, slender, linear, contracted, and sterile; the fifth occurs as a petal with adjoined anthers; the style is of the same length of the filament with a simple hooked stigma. The ovary is roundish, 3-loculed, and contains numerous small dehiscent seeds (Nair 2019; Flora of China 2025).

The rhizome is shallow, thick, fleshy, and tan-colored on the outside with scaley annulated rings (remnants of leaf scars); the inner color of the rhizomes is a brilliant orange-red. The rhizomes have a distinct earthy aroma and have specialized oil cells (Woodville 1792; Ravindran et al. 2007; Flora of China 2025) (see Figure 1(a–d)). The macro- and micromorphological characters of *Curcuma* species with rhizomes have unique characteristics that allow for discernment to species based on shape and size of the mother rhizomes and lateral branches, color and characters of the cross section, aroma, and taste (Seema 2015; Anu et al. 2020).

**Figure 1.**
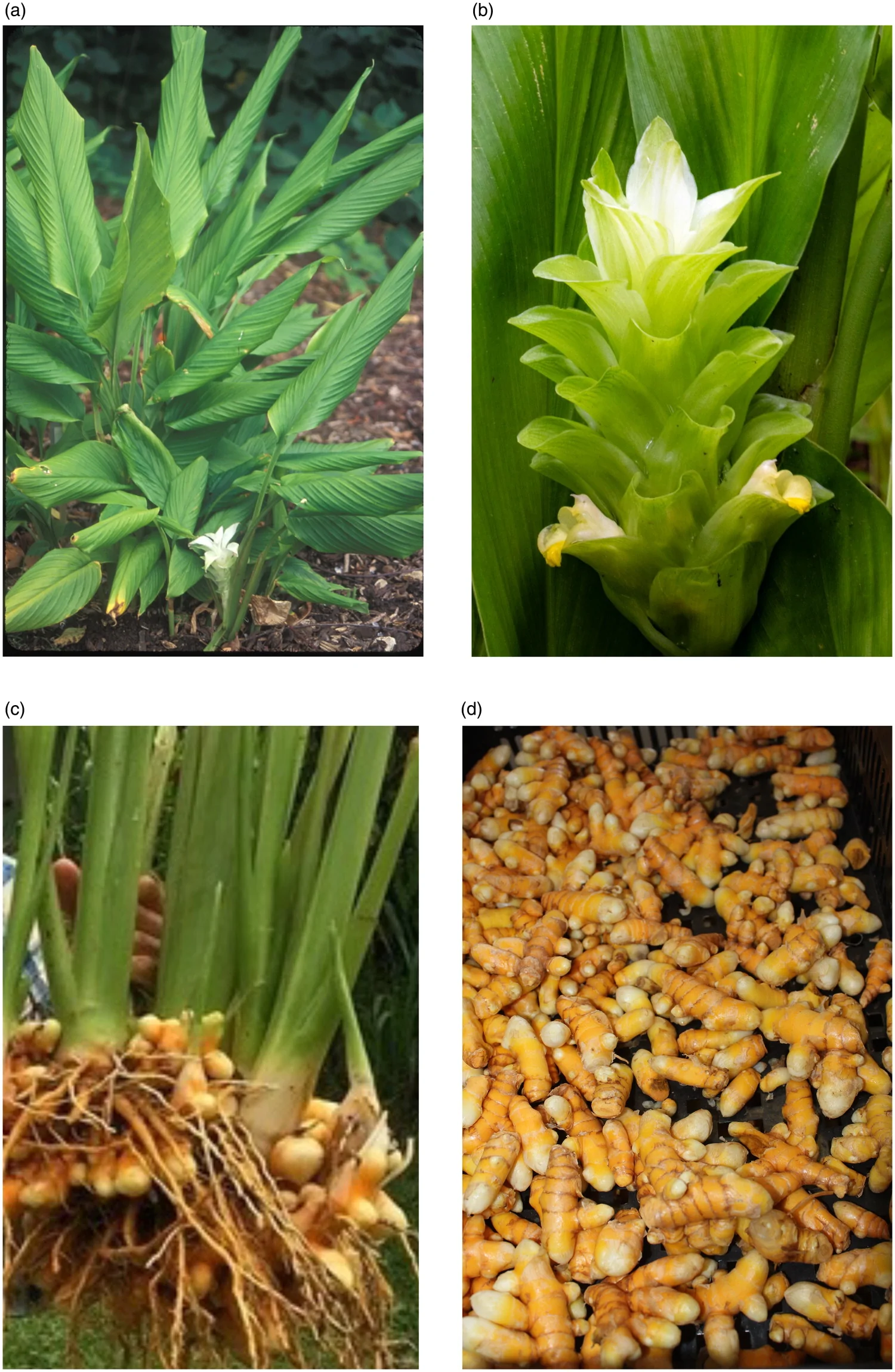
(a–d) Turmeric plant, flower, attached and harvested rhizomes.

**Distribution:** In India, turmeric is available in all states, but particularly in Tamil Nadu, West Bengal, and Maharashtra. It is a tropical crop cultivated at sea level to 1,200 meters predominantly growing in light black clay loam soils and red soils under irrigated and rainfed conditions. Beyond India, turmeric is extensively cultivated in Pakistan, China, Haiti, Jamaica, Peru, Taiwan, Nigeria, and Bangladesh. Other relatively minor producers include Japan, Indonesia, Sri Lanka, Cambodia, Malaysia, Philippines, and the United States (Bejar 2018; Wang et al. 2023), while production in Africa is increasing (Abia et al. 2025).

### Nomenclatural history

The genus *Curcuma* was formally codified by noted Swiss taxonomist Carolus Linnaeus in his *Species Plantarum* of 1753 (see Figure 3), though the name was used prior to this. The name *Curcuma* likely originated from the early Arabic *kurcum*, which means ‘yellow color’ and was Latinized to *Curcuma* (Plowden 1972; Ravindran et al. 2007; Swamy 2025). The species name *longa* means long and refers to the relatively long lateral rhizomes. The origin of the common name *turmeric* is not definitively known but is believed to be derived from Middle English or Early Modern English as *turmeryte* or *tarmaret* or of the Latin *terra merita* (meritorious earth), the meaning of which is unknown. To Dioscorides turmeric was referred to as *Cyperus indicus* (Gerard 1633). Table 2 presents some of the names ascribed to turmeric in India and their meanings, which provide an indication of both the uses and reverence held for turmeric in Indian culture.

**Table 2.** Names assigned to turmeric in India and their meanings.

Anestha (not offered for sacrifice or homage)	Bhadra (auspicious or lucky)
Bahula (plenty)	Dhirgharaja (long in appearance)
Gandhaplashika (which produces good smell)	Gauri (to make fair)
Gharshani (to rub)	Haldi (that draws attention to its bright color)
Haridra (dear to hari, Lord Krishna)	Hemaragini (exhibits golden color)
Hridayavilasini (gives delight to heart, charming)	Jawarantika (that which cures fever)
Jayanti (one that wins over diseases)	Kanchani (exhibits golden color)
Kaveri (harlot)	Krimighni or kashpa (killer of worms)
Kshamata (capability)	Laxmi (prosperity)
Mangalya (auspicious)	Mangalprada (who bestows auspiciousness)
Mehagni (killer of fat)	Nisha (night)
Nishakhya (known as night)	Nishawa (clears night and imparts color)
Patwaluka (perfumed powder)	Pavitra (holy)
Pinga (reddish-brown)	Pinja (yellow-red powder)
Pita (yellow)	Pitika (which gives yellow color)
Rabhangavasa (which dissolves fat)	Ranjani (which gives color)
Ratrimanika (as beautifiul as the moon)	Shifa (fibrous root)
Shiva (gracious)	Shobhna (brilliant color)
Shyama (dark colored)	Soubhagaya (lucky)
Survana (golden color)	Survanavara (which exhibits golden color)
Tamasini (beautiful as night)	Umavara (Parvati, wife of Lord Shiva)
Vairagi (who remains free from desires)	Varna datri (enhancer of body complexion)
Vishagni (killer of poison)	Yamini (night)
Yoshitapriya (beloved of wife)	Yuvati (young girl)

Source: Modified from Swamy 2025.

### Macroscopic identification

Turmeric rhizomes are very distinctive and characteristic. They can be divided into two segments; the central portion (mother rhizome) is ovate to oblong or pyriform (pear-shaped), to round and is known as ‘round turmeric’; the lateral axillary branches or secondary rhizomes are referred to as ‘fingers’ or ‘long turmeric’ and are often short-branched of varying lengths and diameters.

‘Round’ turmeric is derived from the swollen internodes arising from the underground leaf-buds. These are oblong, ovate, or pear-shaped, 3–5 cm long and 3 cm thick and somewhat flattened, about half as wide as long. **‘**Long turmeric is derived from the less fleshy underground branches. This portion of the rhizome is cylindrical, spindle- or finger-shaped, tapers bluntly at each end, and is typically short branched, 2–5 cm long and about 1–2 cm thick.

The natural coloring of the outer surface of both types is light or dark yellowish-brown, smooth and irregular, typically marked with longitudinal wrinkles and transverse rings of leaf scars, and sometimes bears large circular scars where knoblike branches have broken off. The inner surface is a brilliant reddish-orange color (Nair 2019). The coloring of processed, par-boiled, or treated rhizomes can vary greatly from a reddish-orange to brilliant yellow (see Figures 2(a,b), 3, 4, 5(a–h)).

**Figure 2.**
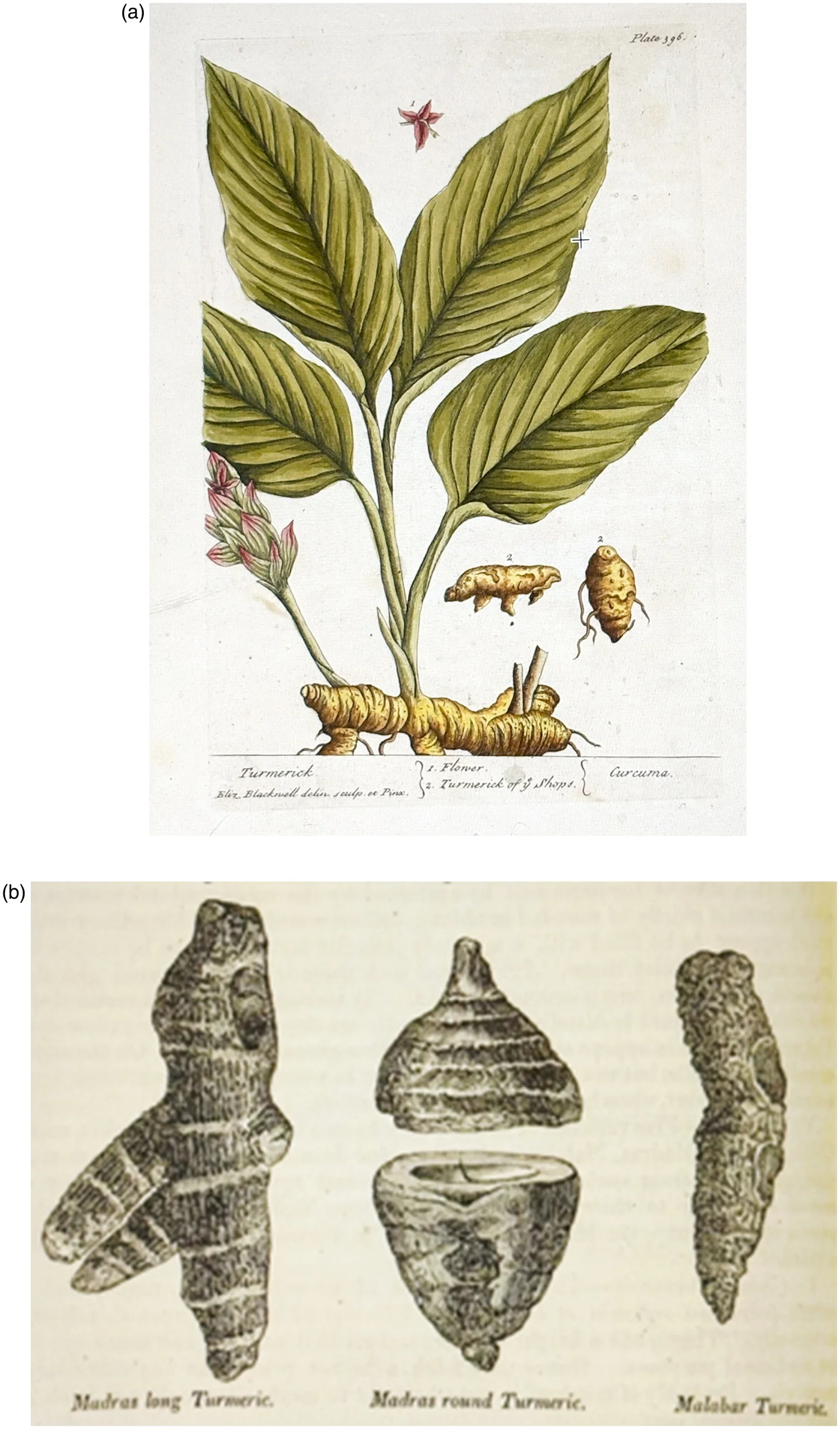
(a) Turmeric illustration Elisabeth Blackwell’s *Curious Herbal* (1737). (b) Illustration of lateral (long) and mother (round) rhizomes (Pereira 1855).

**Figure 3.**
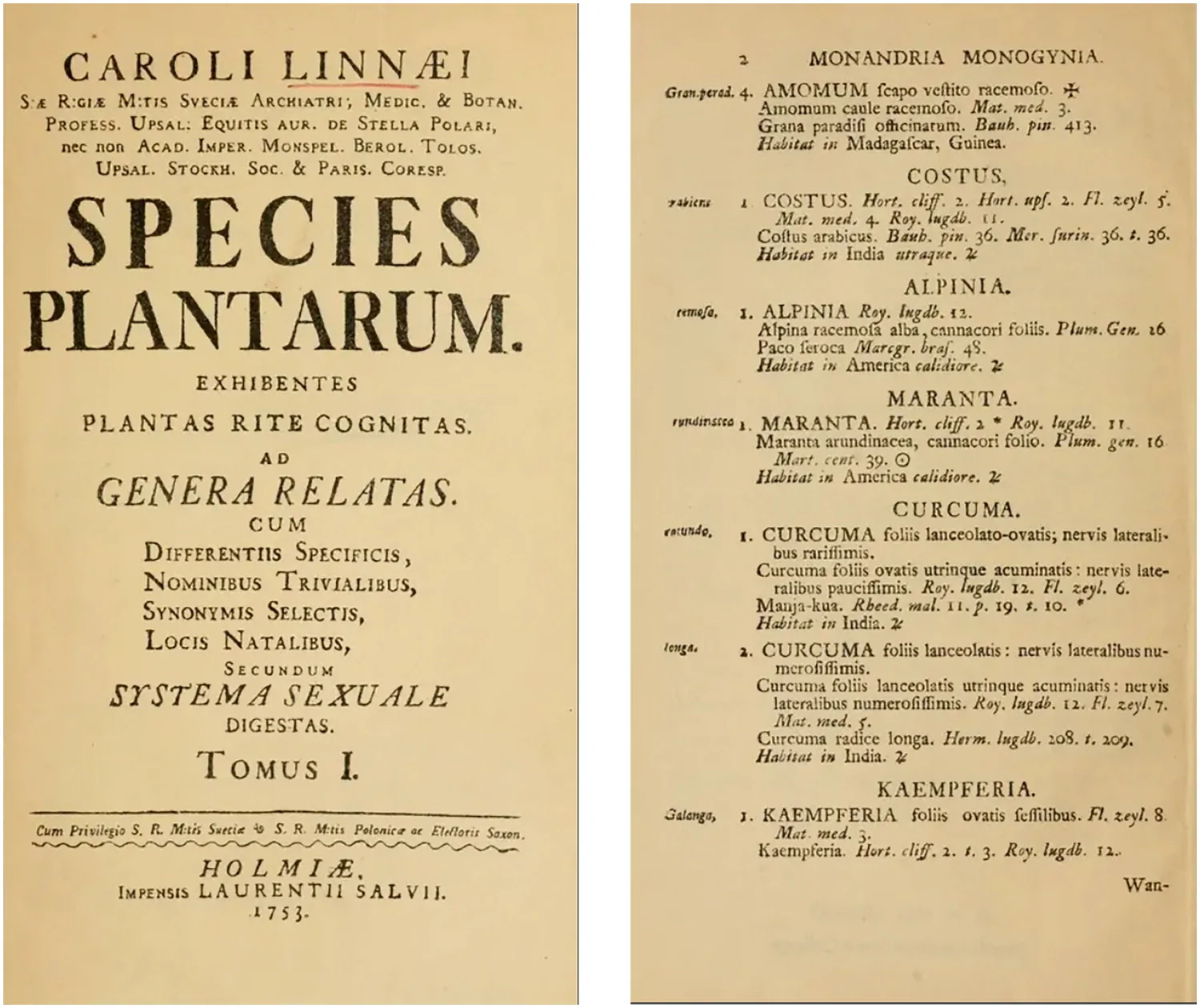
Title page and turmeric entry of *Species plantarum* of Swedish botanist Carolus Linnaeus firmly establishing the nomenclature *Curcuma.*

**Figure 4.**
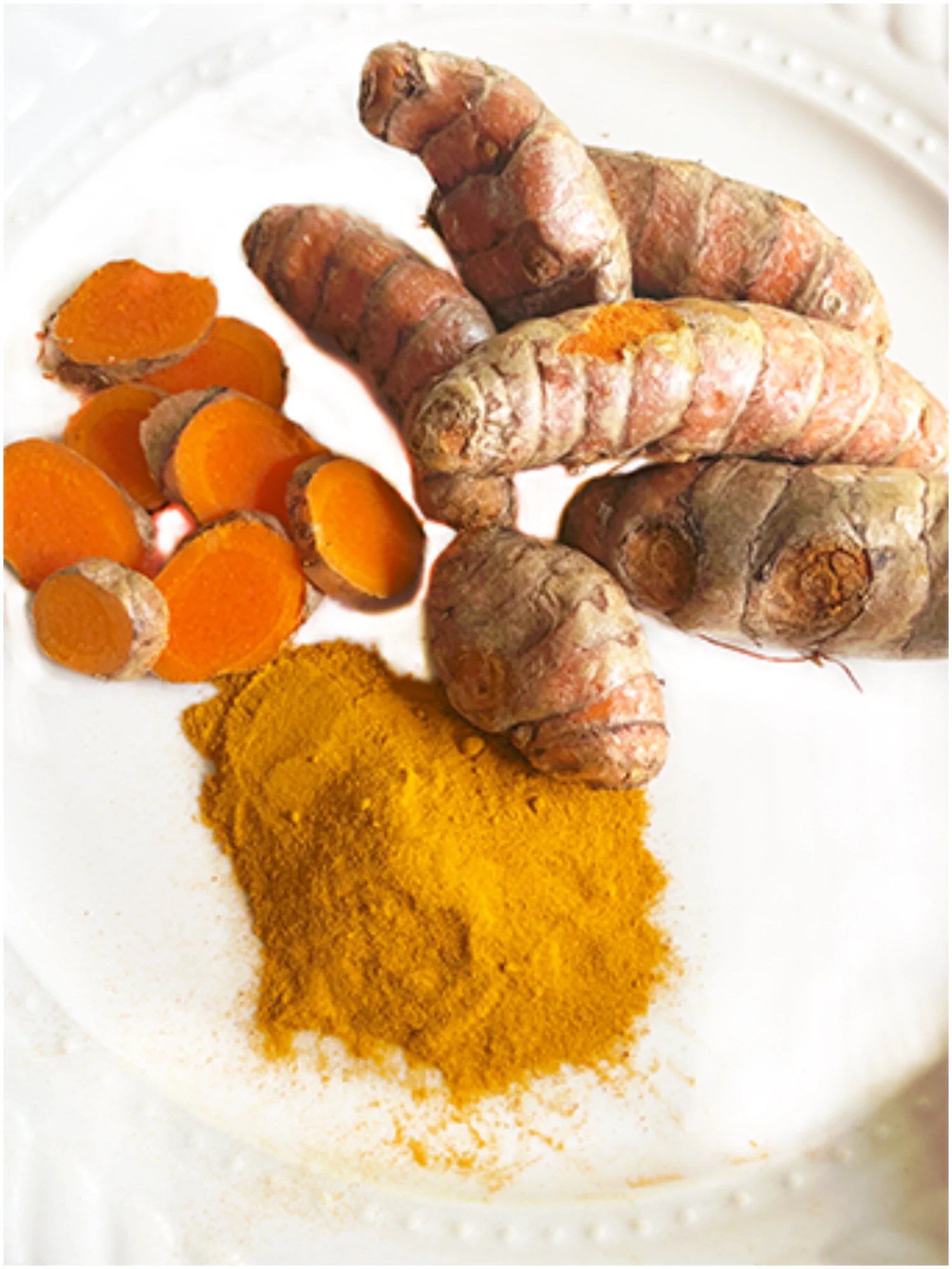
Turmeric rhizomes, powder, and slices.

Figure 5.(a–g) Fresh and dry rhizomes: (a) whole fresh lateral (long); (b) mother (round) and lateral (long); (c) rhizome whole and cross cut of fresh lateral rhizomes; (d) unpolished rhizomes; (e) polished (top) and unpolished rhizomes (bottom); (f) longitudinal slices; (g) cross section of yellow turmeric.

**Figure:**
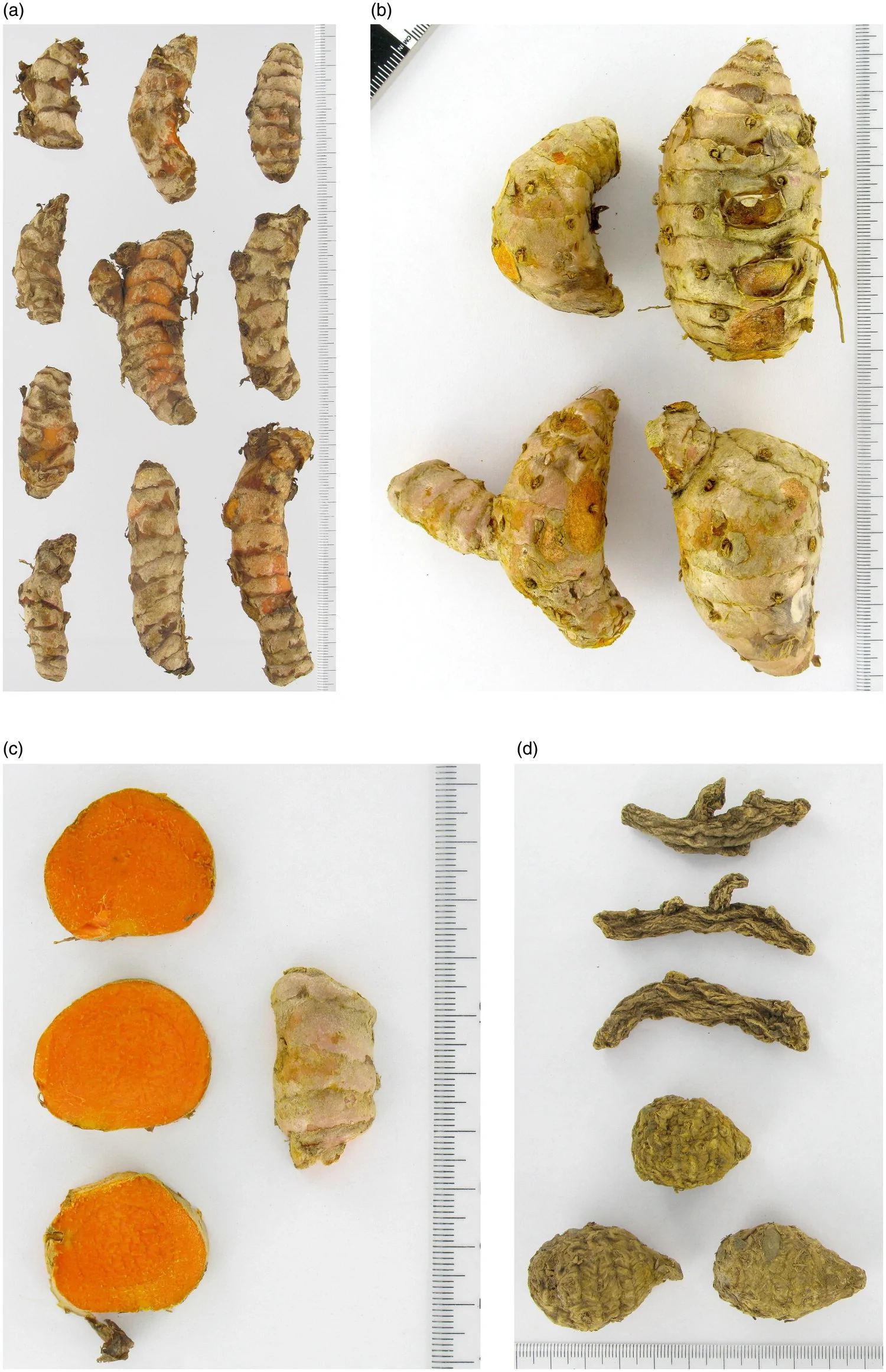

**Figure:**
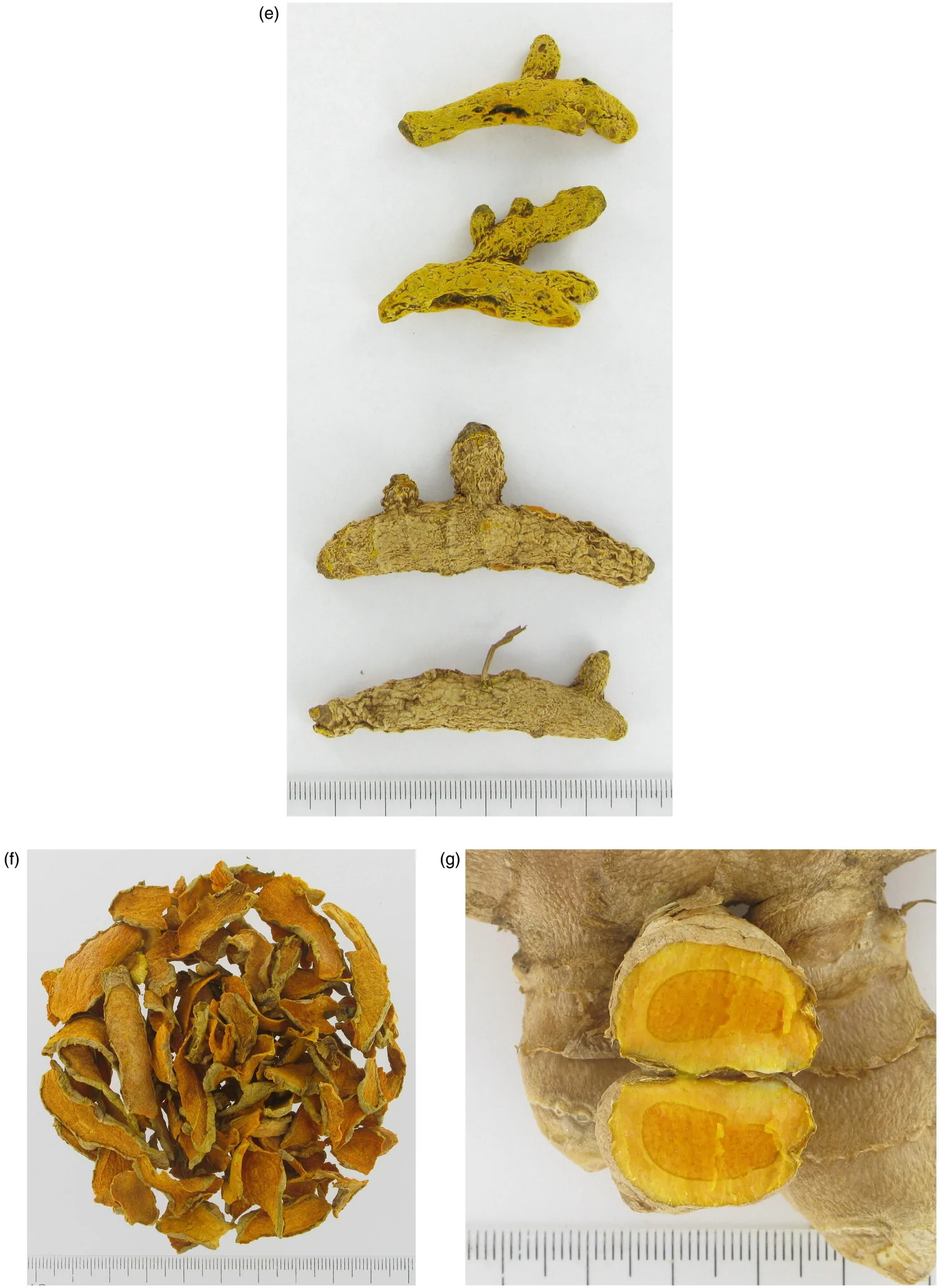

### Chemosensory characteristics

**Color:** Fresh rhizomes are reddish-yellow to brown on the outer surface. Dry rhizomes are brown to buff grey when unpolished and can be more yellow when polished or treated with lead chromate.

**Odor:** Characteristic, aromatic, sometimes described as ‘ginger-like’ for both fresh and dry turmeric.

**Taste and mouth feel:** Characteristic, warmly aromatic, acrid, and bitter; when chewed, the saliva is distinctly yellowed for both fresh and dry turmeric.

**Powder:** Dry turmeric is deep yellow to orange-yellow, faintly aromatic, somewhat bitter and acrid.

**Texture:** Fresh rhizomes are fleshy and somewhat soft but dense. Whole dry rhizomes are hard and snap upon fracturing. If unpolished, the rhizome surface is rough; if polished, the rhizome surface is relatively smooth.

**Fracture:** The fracture of dry rhizomes is short, dense, solid, and granular but fine textured and shows a circular nucleus sheath with numerous vascular bundles; the inner surface is orange-yellow to reddish-brown and somewhat shiny or waxy; the central vascular cylinder (stele) is twice as wide as the cortex; a pale or sometimes dark ring of epidermis separates the central cylinder from the cortex (Maisch 1892; Felter and Lloyd 1898; CPS 1911; Kraemer 1920; Wall and Suppan 1928; Wallis 1946; API 1989; Kapoor 1990; Wichtl and Bisset 1994).

### Microscopic identification (Figure 6(a–g))

**Surface view:** Polygonal epidermal cells; uni- or bicellular, thick-walled covering trichomes, tapering, up to 400 µm long.

**Transverse section:** The transverse section is mostly circular. The epidermis consists of five to six layers of tangentially elongated cells; the sub-epidermal cork layer may contain small calcium oxalate prisms; 15–20 rows of cortical parenchyma of large spheroidal cells, including some secretory cells filled with orangish yellow secretions; small sclereids and scattered vascular bundles are found occasionally in the parenchyma and cork; collateral vascular bundles are scattered in the cortical parenchyma, each consisting of few vessels and a narrow phloem; vessels up to 50 µm diameter; distinct endodermis consists of rectangular, tangentially elongated cells; numerous vascular bundles occur in a ring directly inside the endodermis; parenchymatous stele with scattered secretory cells and vascular bundles similar to those in the cortex.

**Longitudinal section:** Scalariform vessels.

**Starch (uncured rhizomes):** Occasional; simple granules; oblong, elliptic, ovate, or sack shaped, some with a papillary protuberance at the hilum, up to 60 µm in length; an eccentric hilum is situated at the narrower end of the granule very close to the margin; striations run across the grains at right angles to the long axis. **Starch (cured rhizomes):** Mostly gelatinized in boiled material.

**Powder prepared for microscopic examination:** Intensely golden yellow in chloral hydrate suspension; primarily, fragments of parenchyma with only few cells intact showing the clumps of gelatinized starch; few fragments with secretory cells; few fragments of cork and vessels; occasional trichomes.

In a comparison of 15 samples from different regions, a statistical analysis showed that yellow starch cells (intact in uncured samples, clumps of gelatinized starch cells in cured samples), rounded to oval parenchyma with thin slightly irregular walls, fibers, and scattered oil drops were observable in all samples. Intact starch cells are very abundant, mostly simple, flattened, oblong to oval or irregular in outline with a small hilum situated at the narrower end. Additionally, an abundance of large reticulate vessels with regularly arranged rectangular pits were observed in 80% of samples; some vessels showed spiral or annular thickening; calcium oxalate crystals (prisms), rarely clusters, were seen in 78% of samples; pale brown cork composed of thin-walled cells, appearing large and polygonal in surface view, were observed in 64% of samples; and oleoresin was apparent in 29% of samples (Amel 2015). When conducting a microscopic assessment, the absence or occurrence of these variable structures must be considered (Upton et al. 2011; Amel 2015; Aarathi et al. 2023).

**Figure 6.**
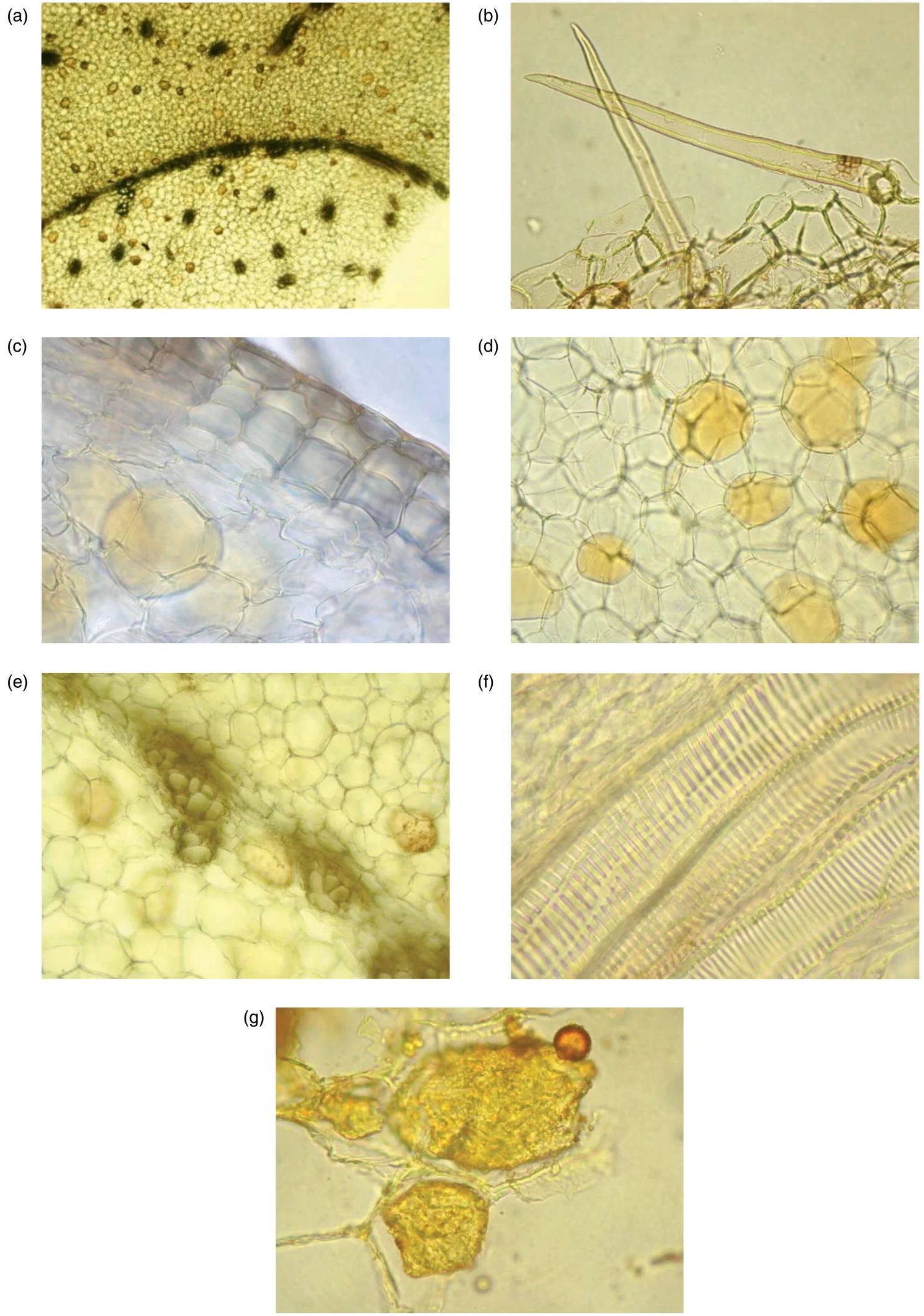
(a–g) Microscopic characterization of *Curcuma longa* rhizome. Source: Upton et al. (2011) with permission of the American Herbal Pharmacopoeia.

### Post-harvest processing

After identification, post-harvest practices are most important for ensuring the quality of raw materials used in the manufacture of finished products. Comprehensive reviews of turmeric adulteration are available in the literature (see Cardellina 2020; Gafner et al. 2026, among others). Following is a brief review of the primary concerns regarding quality and adulteration.

Typically, the lateral portions of the rhizome are separated from the round portion and cleaned of adhering dirt and sometimes broken into smaller pieces for ease of drying. In order to reduce drying time, make a product of greater uniformity, and modify the aroma of the raw material, rhizomes are often par boiled (Praditdoung et al. 1996). Rhizomes are placed in pans or earthenware filled with water and covered with turmeric leaves and a solution of sodium bicarbonate (0.05–0.1%). The rhizomes are then boiled for 40–45 min (India) or six hours (Pakistan), depending on the variety and region. Rhizomes are removed from the water, allowed to cool, and immediately dried in the sun. Dry rhizomes may be further processed by polishing to remove the rough surface (Prasad and Aggarwal 2011).

### Adulterants, substitutions, and sophistications

**Other species:** Due to the ubiquitous nature of turmeric and its widespread cultivation, fresh whole rhizomes are generally not subject to adulteration. Mix ups can occur with *C. zedoaria* and *C. xanthorrhiza*, especially when powdered (Sen et al. 1974; Tiwari et al. 2013). *C. zedoaria* may be the most likely species associated with confusion as it looks most like *C. longa*, though the rhizomes are more yellow and contains 10 times more demethoxycurcumin than *C. longa* making morphological and curcuminoid ratios important considerations for species identification (Avula et al. 2012).

**Coloring agents:** During processing, a yellow coloring such as lead chromate or a dye may be used to intensify the yellow coloring of the rhizomes (Dixit et al. 2008; Dhanya and Sasikumar 2010; Sarkar and Ghosh 2012; Tiwari et al. 2013; Cowell et al. 2017). Lead and chromium are inexpensive, are among the heavy metals of greatest safety concern, and have resulted in toxic exposures in children consuming turmeric (Gleason et al. 2014). There are a number of analytical techniques that can detect contamination with metals. Some of these include: colorimetric, laser-induced breakdown spectroscopy, inductively coupled plasma mass spectrometry, x-ray fluorescence spectroscopy, and multi-collector inductively coupled plasma mass spectrometry. Other colorants found in turmeric include Metanil yellow and Sudan red G (Bejar 2018; Cardellina 2020).

**Fillers and excipients:** Unlabeled fillers or excipients, such as gluten-containing flours (e.g., wheat) or allergen-containing materials (e.g., nuts) may be present (Parvathy et al. 2015). While representing a health hazard to consumers with sensitivities, allergies, or other unfavorable reactions to such substances, if undeclared, such products are violative of allergen labeling requirements in some countries. Starchy fillers have been reported in turmeric supplements and include corn (*Zea mays*), wheat (*Triticum aestivum*), rice (*Oryza sativa*), and tapioca (*Manihot esculenta*) (Cardellina 2020).

**Colorimetric test for dyes**: Approximately 1 g of turmeric powder is added to plain water in a clear glass. Authentic turmeric turns the water a brownish color after a few minutes; material to which dye has been added turns the water a brilliant yellow rapidly.

**Extracts and synthetic curcumin:** Curcuminoids are the primary compounds of interest in turmeric and are responsible for the yellow coloring of rhizomes (see Figure 7). Turmeric contains approximately 50 curcuminoids, three of which dominate, namely: curcumin (77%), demethoxycurcumin (17%), and bisdemethoxycurcumin (3–6%); different varieties and growing conditions can yield varying ratios and concentrations of curcuminoids. These three curcuminoids are used for the quality control of turmeric rhizomes and extracts. Raw rhizomes contain approximately 5–9% total curcuminoids, though concentrations of approximately 14% have been reported in fresh rhizomes. Pharmacopeial standards vary but establish a minimum quantity of 1–3% curcumin calculated as the sum of the three primary curcuminoids by liquid chromatography (HPLC) (Paudel et al. 2025). Some manufacturers and researchers give greater focus to another class of potentially clinically relevant compounds known as *turmerones* (Golding et al. 1982; Niknejad et al. 2025).

**Figure 7.**
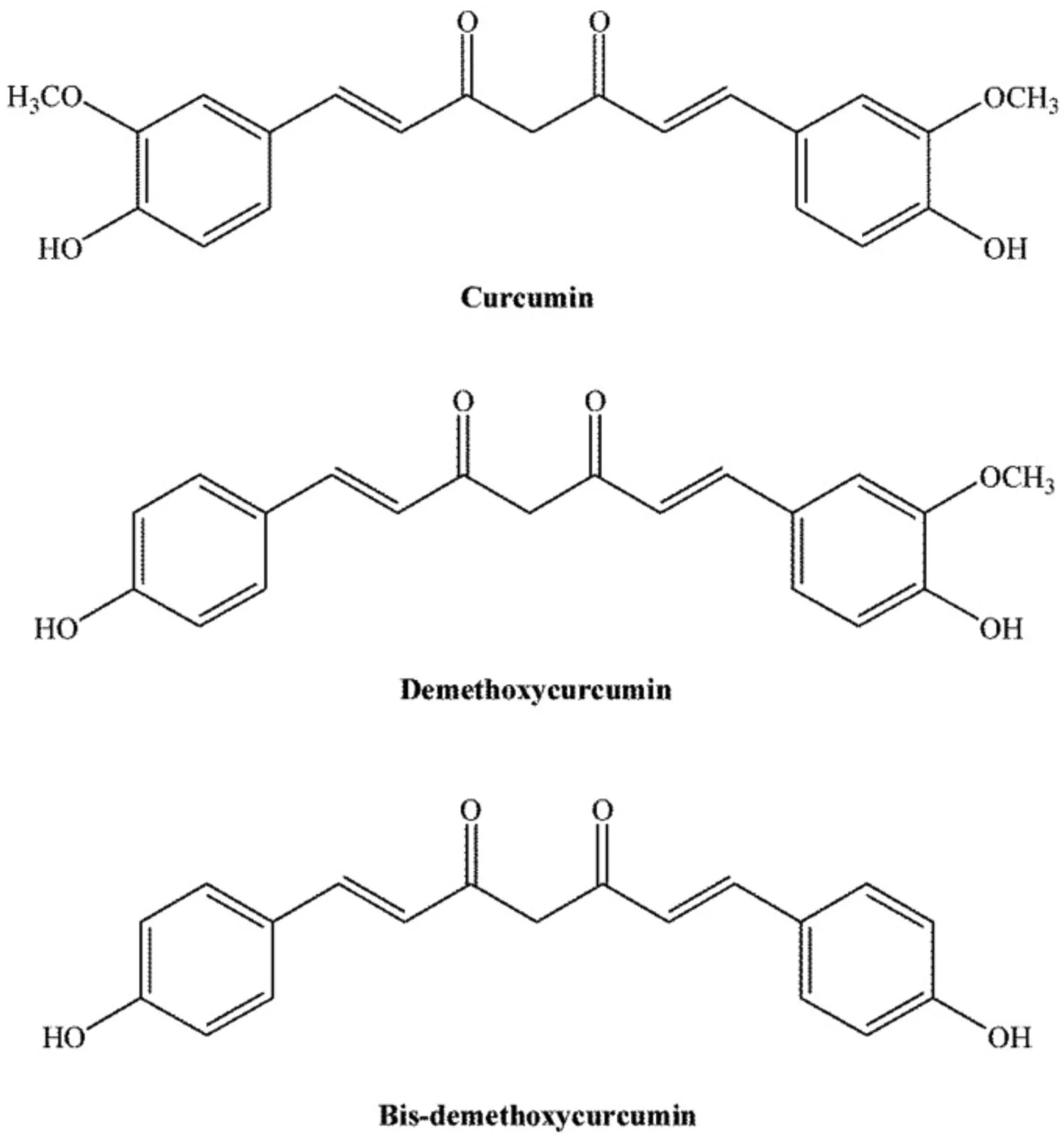
Primary curcuminoids of turmeric.

Due to its biological activity, curcumin is widely synthesized and used in extracts but labeled as turmeric, resulting in adulterated products. There are currently no validated methodologies for detecting synthetic curcumin, though use of mass spectrometry to evaluate ^14^C content of turmeric has been explored (Cardellina 2020). Many extracts are labeled as yielding 95% curcuminoids, which either require the use of organic extracting solvents or use of synthetic curcumin. Samples that are spiked with synthetic curcumin can sometimes be detected chromatographically. With high performance thin-layer chromatography (HPTLC), a partial or complete substitution with synthetic curcumin may be detected by the presence of a single band corresponding to curcumin but lacking or containing disproportionate amounts of desmethoxy- and bisdemethoxycurcumin (Booker et al. 2014). Similar anomalous ratios of curcuminoids are evident with other analytical methods as raw material turmeric has a relative fixed ratio of curcumin to desmethoxycurcumin to bisdemethoxycurcumin of approximately 70–80%, 15–25%, and below 6.5%, respectively. Synthetic curcumin also lacks these two latter curcuminoids (Girme et al. 2020).

### Historical use

Turmeric is one of the most important cultural and medicinal plants of all traditions who had access to it. An extensive review of the historical use of turmeric by world cultures is beyond the scope of this review, but highlighted are some of the primary uses recorded in the ethnobotanical literature with a focus on its use in India. India remains the primary source of turmeric globally and is also the largest consumer of turmeric, where it is used both as a food and medicine. Much of the traditional and formal medical use of turmeric in India is reflective of its use throughout the rest of the world. Following is a brief accounting of the historical and traditional use of turmeric in India, as well as other cultures, which mostly follow the medicinal uses recorded in Indian literature (see Table 3).

**Table 3.** Representative cross-cultural use of turmeric.^a^

Country	Earliest record	Uses	References
Africa	800 CE	Food, colorant, ceremonially, cosmetic; common cold, arthritis, sterility, crying eyes in children, blood sugar control, inflammation, digestive complaints.	Jagganath (2020); Prasad and Aggarwal (2011)
China	700 CE	Food, colorant; inflammatory and chronic diseases, conditions associated with blood stagnation, menstrual pain, pain and swelling due to trauma, joint pain.	Bensky and Gamble (1996); Prasad and Aggarwal (2011)
Europe	thirteenth century	Food, colorant; suppressed menses, jaundice, liver obstructions, digestive, diuretic, kidney stones, gall obstructions	Green (1824); Parkinson (1640); Thorpe (2025)
India	1974 BCE	Food, colorant, ceremonial use; allergies, anorexia, arthritis, asthma, cough, diabetic wounds, flatulence, gallstones, liver disorders, menstrual regulation, rheumatism, runny nose, sinusitis, strengthening tonic, worms.	Prasad and Aggarwal (2011); Scott et al. (2021)
Polynesia		Food, ceremonial use; ‘sovereign remedy for many diseases’, nose, ear, and throat afflictions, inflammation.	Sopher (1964)

^a^Most of the ethnobotanical uses of turmeric recorded in the world’s literature is consistent with use recorded in Indian literature.

#### Archaeological and historical evidence

The use of turmeric appears to extend far into antiquity to almost 2,000 BCE in India and Pakistan, at which time the spice was distributed from its origins in Southeast Asia into the Mediterranean, along with black pepper (*Piper nigrum*) and cinnamon (*Cinnamomum* spp.) (Young 2001; Prance and Nesbitt 2012; Van der Veen and Morales 2015; Wang et al. 2023). In addition to this fossil record, turmeric is mentioned in Assyrian medical texts from Ashurbanipal’s library at Nineveh (seventh century BCE) (Wang et al. 2023).

### Traditional uses and cultural significance of turmeric in Southeast Asia

In addition to its ubiquitous use in food and as a dye, turmeric is of great significance in all aspects of Indian and other South Asian cultures from birth to death, marriage, personal decoration and adornment, textiles, economies, ceremonial and religious use, worship, and magic. Historically in India, human sacrifice (Meria sacrifice) was practiced to ensure successful turmeric crops, a practice outlawed by the British in 1845 and replaced with the sacrifice of a buffalo, a persistent practice in some regions today. The primary use of turmeric according to one review was as a substance of magic in religious rites; second as a Sakthi worship; then as cosmetic, food, and medicine (Velayudhan et al. 2012).

In Hindu culture, turmeric is regarded as a substance of purity, thus its use in religious ceremonies. When a baby is born, its body is anointed with a paste made from fresh turmeric with the belief this will promote a long life (Velayudhan et al. 2012). In parts of Indonesia, after birthing, the mother and child may be massaged with turmeric, a midwife may chew a piece of rhizome and then spit over the baby as a blessing, or a piece of rhizome may be buried with the umbilical cord (Sopher 1964). Also in Indonesia, turmeric is regarded as a galactagogue. Nursing mothers use it to reduce pain, lower blood pressure, as an anti-inflammatory, and to increase blood circulation (Sayuti and Atikah 2023). In Central Java, new mothers often consume approximately 200 mL of a turmeric infusion every day for one week after delivering (Sumarni and Anasari 2019).

In India, during some festivals (Raja Sankranti for sowing or Raksha Bandan for protection of sibling relationships), boys and girls anoint their bodies with turmeric paste or animals may be anointed. Some women on the last day of the menstrual period apply turmeric paste before bathing to purify themselves so that they may return to a routine domestic life and regain touchability. This is considered an ancient practice, especially among Brahman communities.

Historically, the use of turmeric as part of marriage ceremonies was widespread wherein the bodies of bride and groom are anointed with a turmeric paste, or a bride may bathe in turmeric water, or employ some other practice. In the late 1800s, it was stated ‘no Hindu girl in the Central Provinces can be married without having her body rubbed with turmeric’ (Russell and Hiralal 1916). Turmeric powder, paste, or solution is commonly used throughout India to welcome newlyweds to their home (Velayudhan et al. 2012).

Turmeric was historically also prominent in preparation of the deceased by either anointing the corpse with a turmeric preparation, sprinkling the grave with a turmeric solution after burial to quiet the spirit of the deceased, or even rubbing the exhumed bones with a preparation of turmeric and coconut oil to honor the dead, thus factoring into every aspect of Southeast Asian culture (Sopher 1964).

In an ethnobotanical review conducted in the Champhai district of state of Mizoran in Northeast India, turmeric was reported as the plant with the highest cultural and use value. Medicinally, it was used for ulcer, diarrhea, skin care, and stomachache (Laldingliani et al. 2022). In a review conducted in the Terai Forest region of Western Nepal others in the Philippines and Thailand, turmeric was similarly the most frequently and popularly used medicinal plant species in the regions. Specific uses included a decoction given as a stimulant, tonic, and blood purifier, for cardiovascular, gynecological, and respiratory care, relief of musculoskeletal or joint pain, fevers, treatment of peptic ulcers and intestinal infection. The juice of the fresh rhizome is given as anthelmintic, and the powder is given orally with warm water for jaundice and liver disorders. Topically, the paste is applied to strains, wounds, and even fractures (Singh et al. 2012; Cordero et al. 2021; Junsongduang et al. 2025).

There is widespread and numerous traditional use of turmeric in India. Some of the primary and most consistent of its internal uses is as an anthelminthic, anti-parasitic, aperient, appetite stimulant, antiseptic, bruises, digestive aid, expectorant, general health purposes, liver (jaundice), improving the complexion, laxative, and as an ingredient in rasayana (longevity) formulas. Topically it is used for a host of skin conditions that include abscesses, bites, boils, burns, itching, ringworm, scabies, snake bite, ulcerated tissues, wounds, and conditions such as, chickenpox, measles, and smallpox. Turmeric is also used as an eyewash for conjunctivitis and burned on a piece of charcoal and inhaled for mucus congestion and sinusitis. Turmeric ointments are regarded as highly effective for treatment of hemorrhoids. For these uses, it can either be used alone or combined with other botanicals. For example, the combination of turmeric with neem leaves (*Azadirachta indica* A. Juss) is very common when used as an antiseptic or for parasites. Dry rhizomes are usually powdered and commonly consumed directly with a little warm water, as tea (infusion or decoction), cooked in scalded milk, consumed with ghee (clarified butter), or taken with jaggery or molasses (Velayudhan et al. 2012).

## Use in formal medical systems

### Ayurveda

India has perhaps the most extensive medicinal use of turmeric. Some authoritative reviews record the therapeutic use of turmeric as occurring in Vedic texts and scripture that predate the formal establishment of Ayurveda as a health and medical discipline. Records of traditional use suggest that consumption of turmeric powder with honey can enhance memory, and, when mixed with ghee, counteracts snake bite (Velayudhan et al. 2012; Nair 2019). Another record suggests that patients suffering from cardiac diseases and jaundice should consume rice mixed with turmeric, while applying turmeric externally over the entire body (Nair 2019).

Turmeric is extensively recorded in the *Caraka Samhita,* the foundational text of Ayurveda dating somewhere around the 2nd to 4th centuries (Trikamji 1992). Alone, turmeric is classified among bitter drugs to be used for enemas. Because of its bitterness, turmeric is considered beneficial for enhancing the appetite, promoting bowel movements, facilitating the flow of bile, and for the treatment of parasites. It is used for jaundice, constipation, loss of appetite, and abdominal fluid. It is also used to relieve pain, promote circulation, treat anemia, stop bleeding, used as an expectorant, and internally and topically employed for a myriad of skin afflictions, including for bites, complexion, wounds, eruptions, and itching (Nair 2019). Additionally, turmeric is combined with other ingredients for almost any indication that can be imagined.

In ayurveda, a plethora of preparations are used that include water or milk infusion, medicated ghee, oil, wash, fomentation, lotion, ointment, enema, paste, eyewash, and inhalation (Sharma 2003; Velayudhan et al. 2012; Nair 2019).

### Traditional Chinese medicine (TCM)

Turmeric was not listed in the foundational text of Chinese herbal medicine *Shennong Bencao Jing* (∼third century) but is included in the monumental *Bencao Gangmu Jing* of Li Shizhen (Li 1596) which records turmeric’s use for abdominal and epigastrium stagnation, relief of belching, and blood stagnation, and the treatment of arthralgia. Today in TCM, turmeric is classified among herbs to invigorate the blood and is used to relieve chest, abdominal, and menstrual pain, pain due to trauma, to regulate the menses, and for arthritis (Bensky and Gamble 1996). The *Pharmacopoeia of the People’s Republic of China* approves of turmeric for stabbing pain in the chest and abdomen, dysmenorrhea, amenorrhea, abdominal masses, aching pain in shoulder and arm, and swelling and pain caused by traumatic injuries (PPRC 2015). In China, turmeric rhizomes is formally regarded as a botanical that can be used both as food and medicine.

### Traditional Persian medicine

Turmeric is featured in the *Canon of Medicine* of noted Persian physician Ibn-Sina (Avicenna) (930–1037) in which it is described as heating and drying. This seminal text records uses of turmeric for jaundice, the skin, digestion, treatment of nerve injury, mania, and improvement of eyesight (Bakhtiar 2012). Turmeric is included in the *Iranian Herbal Pharmacopoeia* (Dehkordi et al. 2003).

### Traditional use in western herbal medicine

The history of western herbal medicine formally begins with the first century *De Materia Medica* of Pedanius Dioscorides of Greece, which remained the primary authority of materia medica for almost 1,700 years. While the text does not explicitly list turmeric, it does list κύπειρος ινδικός (*Cyperus indicus*), which was described as a ‘form of ginger that, when chewed, colors the saliva yellow like saffron’. This, Gerard (1633) in his *Herbal* attests ‘without a doubt’ is *Turmerick*. No medical uses were recorded by Dioscorides (Aboca 2013). Thereafter, it appears that turmeric was not prominently featured in early Western herbal medicine traditions. It reportedly occurred on a list of drugs sold in Frankfurt, Germany in 1450 and another inventory list of 1578 (Fluckiger and Hanbury 1879).

Despite its inclusion in early medicament inventory lists, turmeric was lacking in early authoritative works such as the *Dispensatory of Valerius Cordus* (1546), which was the forerunner to all formal Western pharmacopoeias. Turmeric was absent from some (e.g., Travagino 1767; Hamilton 1792) and listed in other Renaissance herbals, where it was sometimes listed as either *Cyperus indicus* (Gerard 1633) or *Crocus indicus* (Salmon 1710). Turmeric is included in the French medical literature in *Histoire Generale des Plantes* (Dalechamp 1653), *Dictionnaire Universel des Drogues Simples* (Lemery 1760), and *A Complete History of Drugs* (Pomet 1768). In these texts, turmeric is only briefly mentioned or simple descriptions of the plant and rhizome are provided, with scant reference to the traditional Eastern medical use or turmeric in foods, as a dye, and for jaundice. One historical record of 1879 reported: ‘in Europe, it has always been less appreciated than the closely related ginger’ and ‘It is obsolete and largely used as a dye’ (Fluckiger and Hanbury 1879).

Some American and English medical literature of the 18th and 19th centuries provide records of turmeric but reflects no medical use beyond its coloring of other preparations, as a color test to detect alkalies, and that it was rarely used (Lewis 1791; Coxe 1818; Edwards and Vavasseur 1829). Turmeric was listed in the first six editions of the United States Pharmacopoeia (1820–1870) predominantly on the secondary list (Boyle 1991) and it was similarly included in early editions of the *British Pharmaceutical Codex* (CPS 1911) that included tincture of turmeric as a coloring agent. A relatively comprehensive accounting of the pharmacognosy of turmeric emerges in 1903 Germany in *Hager’s Handbuch der Pharmaceutischen Praxis* (Fischer and Hartwich 1903) and thereafter, interest in turmeric grew in the west. Turmeric was named 2026 Herb of the Year by the International Herb Association (Readal 2026). It is one of the most widely researched botanicals in the world with more than 9,000 references to ‘turmeric’ listed in Web of Science as of 2026.

### Inclusion in pharmacopoeias and authoritative works

Turmeric is recognized broadly in contemporary pharmacopeial monographs and compendial works, including the German Commission E, United States Pharmacopeia, British Pharmacopoeia, European Pharmacopoeia, Mexican Herbal Pharmacopoeia, Japanese Pharmacopoeia, Korean Herbal Pharmacopoeia, Pharmacopoeia of the People’s Republic of China, Ayurvedic Pharmacopoeia of India, Unani Pharmacopoeia, Siddha Pharmacopoeia, and Taiwan Herbal Pharmacopoeia, to name a few, as well as reviews of the World Health Organization, European Medicines Evaluation Agency (EMA 2018), and European Scientific Cooperative on Phytotherapy (ESCOP 2003).

In evidence-based reviews globally, the following actions, uses, and indications for turmeric have been formally approved by different medical authorities. Treatment of peptic ulcers, pain and inflammation due to rheumatoid arthritis, dysmenorrhea, diarrhea, epilepsy, pain and skin diseases, asthma, boils, bruises, coughs, dizziness, and insect bites (WHO 1999). Clinical studies demonstrate that turmeric influences bile, pancreatic, and gastric secretions and is used for treatment of acid, flatulent, or atonic dyspepsia (Blumenthal et al. 1998; WHO 1999), has anti-inflammatory and choleretic activity, and can be used as a digestive aid for the symptomatic treatment of mild digestive disturbances and minor biliary dysfunction (WHO 1999; ESCOP 2003)^,^. A formal review of the European Medicines Evaluation Agency concluded that crude turmeric preparations can be used for relief of mild digestive problems, such as feelings of fullness, slow digestion, and flatulence (EMA 2018).

## Conclusions

Turmeric is one of the most important herbs of all traditional cultures who had access to it historically. Its primary use is informed from both local and formal medical practice in India, which provides most of the world’s population with the rhizomes. The familiarity with turmeric as a primary spice used in curries is almost universal. The use of turmeric as a dye plant is ubiquitous throughout Southeast Asia, where in addition to its use for providing yellow color to the robes of monks is also used culturally and ritualistically in the most auspicious of ceremonies that include birth, marriage, death, agriculture, worship, and more. While turmeric was introduced to the Western world throughout the past several centuries, it was only in recent decades that its medicinal activity has gained respect, predominantly through the fact that it is one of the most widely used botanicals in Southeast Asia and that it is also one of the most comprehensively researched botanicals in the world.

Traditionally in Ayurvedic medicine, alone, it was predominantly used to dispel stagnation and obstructions in all organ systems, mainly in the gastrointestinal tract, but also for the circulatory, respiratory, and renal systems with the treatment of jaundice being one of its primary indications. In combination with other botanicals, it is considered a beneficial ingredient for treating almost any condition. Various preparations of turmeric are used both internally and topically, and include infusions with water or milk, powders, medicated ghee, medicated oils, ointments, lotions, eyewashes, paste, and enemas. Few other botanicals are used in as broad of a fashion. Turmeric was extensively used topically, similarly for myriad of skin afflictions, though the yellow staining power of the rhizomes may preclude its use by some. Most therapeutic uses by other cultures has predominantly been informed by its use in Southeast Asia and its use in traditional healing systems is very consistent with uses such as in India.

Various species of *Curcuma* are in global trade. Because *C. longa*, the source species for turmeric, is widely cultivated, there is a little risk of adulteration when trading whole material. However, once powdered, there is the potential for other species to be mixed in. Conversely, there are robust methodologies ranging from gross morphology, sensory characteristics, microscopic structures, chemical differences, genetics, and adulteration tests for ensuring authenticity, purity, quality, and potency. These are presented by national and international standards setting organizations (e.g., European Pharmacopoeia, Indian Pharmacopoeia, International Standards Organization, Pharmacopoeia of the People’s Republic of China, United States Pharmacopeia, etc.).

The most significant adulteration which poses a potential public health risk is the presence of colorant agents to enhance the yellow color, the most potentially dangerous being lead chromate, the addition of which is a common practice by many producers. Another quality concern is dilution due to the presence of a fillers like starch or the presence of other coloring agents. For extracts, the most significant concern is the marketing of synthetic curcumin without disclosure of its presence in products marketed as turmeric. Such non-disclosure renders products illegal or adulterated under most federal laws. Currently, robust scientific methods for distinguishing between synthetic and naturally derived curcumin are not available but may be indicated by anomalous concentrations and ratios of curcumin to other curcuminoids.

In recent decades, there have been numerous evidence-based reviews conducted by international medical authorities who predominantly support the use of turmeric and its preparations for the primary indications for which it was used, primarily for digestive and intestinal disorders. This review provides the ethnobotanical basis for the use of turmeric for this special edition. Other contributions will focus on chemistry, pharmacology, analytics, therapeutics, and safety.

## Data Availability

All resources used are readily available through databases, online, or library sources and can be provided upon request.
